# A comparative analysis of adolescents' emotions and emotion regulation strategies when witnessing different cyberbullying scenarios

**DOI:** 10.1016/j.heliyon.2024.e29705

**Published:** 2024-04-17

**Authors:** Sónia da Silva Gomes, Paula da Costa Ferreira, Nádia Pereira, Ana Margarida Veiga Simão

**Affiliations:** CICPSI, Faculdade de Psicologia, Universidade de Lisboa, Portugal

**Keywords:** Emotion, Emotion regulation, Cyberbullying, Prosocial behavior, Sex differences

## Abstract

The search for greater popularity and acceptance by peers increases the use of social networks that may cause cyberbullying. The high number of adolescents who observe this phenomenon may help reduce the negative impact on the victims. Emotion regulation is an important predictor of adolescents' psychological adjustment and social competence to adopt a prosocial behavior. Bystanders of these incidents may misinterpret what they see, due to specific cyberbullying characteristics which may influence emotion regulation negatively. Studies about emotions and emotion regulations in bystanders are scarce. Therefore, this study aims to investigate the emotional reactions and emotional regulation strategies of bystanders while witnessing various cyberbullying scenarios (posting photographs, direct threats, offences to integrity, threats to share personal information), as well as to focus on different behavior, victim characteristics, aggressor traits, and spectator reactions. A convenience sample of 143 adolescents (from 12 to 17 years old) was used. Results showed that worry and sadness were the most expressed emotions in all scenarios (mainly for girls) and posting a photograph was considered more concerning for girls and boys both. To regulate emotions, participants used distraction (especially seven graders) and rumination strategies. Nonetheless, when posting a photo without permission, they all adopted prosocial behavior (boys and girls) because they considered it more serious. Seventh graders adopted less prosocial behavior than eighth graders. In short, girls were more prosocial than boys. Boys may have more difficulty in regulating emotions properly and it may contribute to not intervening. Also, younger adolescents may have experienced less emotional maturity. Therefore, developing programs based on socio-emotional skills, which increase awareness of the seriousness of cyberbullying, can teach youth how to deal with emotions in order to regulate them effectively, thus increasing emotional maturity.

## 1 Introduction

### 1.1 Cyberbullying, its impact and associated factors

Cyberbullying, defined as aggressive and intentional acts using electronic devices, has escalated globally, particularly among adolescents aged 11 to 18, with a surge during the pandemic [[1], [2], [3], [4], [5]]. This alarming trend has raised concerns due to its negative impacts on socio-affective development, academic performance, and overall well-being [6,7].

Researchers have identified predictors and perpetuating factors of cyberbullying, including age, sex, internet addiction, impulsivity, lack of empathy, and deficits in emotional regulation [[8], [9], [10], [11], [12], [13], [14]]. Understanding these factors is crucial for effective intervention [15]. This study focuses on age, sex, and emotion regulation strategies among bystanders, aiming to shed light on their role in cyberbullying prevention [[16], [17]].

Bystanders play a significant role, providing social support to victims, with perceived support acting as a protective factor against cyberbullying [[16], [18], [19]]. Social support is viewed as a coping mechanism to withstand stressful situations [20]. However, challenges in emotion regulation may increase the risk of cyberbullying, emphasizing the need to understand how bystanders regulate emotions and why they may hesitate to intervene [21,22].

The characteristics of cyberbullying, such as anonymity, physical distance, and role overlap, contribute to its occurrence [23]. Anonymity allows for aggressive behavior without identification, leading to disinhibition and antisocial tendencies [24]. Physical distance in the virtual world hinders emotional connection with the victim, making it difficult to interpret emotions and cues, potentially resulting in inadequate responses [25,26].

Different forms of cyberbullying exist, influencing bystanders' perceived severity of the phenomenon [27,28]. Verbal aggression and image appropriation are prevalent, with varying degrees of severity attributed to different forms of aggression [29,30]. This study aims to identify which aggressive behaviors adolescents consider more serious, guiding interventions to foster prosocial behavior.

The influence of age and sex on cyberbullying remains a topic of investigation. Incidents peak between ages 13 and 15, with older students (15–18 years old) at higher risk [14,31]. Conflicting findings on the influence of sex highlight the complexity of cyberbullying dynamics, with impulsivity as a risk factor and empathy as a protective factor [15,32,33]. This study emphasizes the need to explore the influence of age and sex on bystanders, including their emotions and regulation strategies, for future interventions [11,14].

### 1.2 Emotions in cyberbullying

The study of emotions dates back to the 19th century with Wundt [34], Watson [35], Lazarus [36] and its research peaking in the 90s. Some theorists even considered emotions to be indispensable for adaptive behavior [37]. Perhaps the best-known modern basic emotions theory was proposed by Ekman [38]. According to Ekman, there are at least six basic emotion modules: joy, sadness, anger, disgust, fear, and surprise. When activated by suitable perceptions or appraisals, emotion-specific feelings and physiological reaction patterns are generated, and an involuntary tendency to show a particular facial expression may occur.

In cyberbullying, emotions play a crucial role, considering that this type of aggression impacts the emotions, behavior, and emotional well-being of adolescents. Emotions have been defined as subjective and idiosyncratic reactions that arise from attention and the subsequent evaluation of a given situation [39,40]. Emotions are composed of physiological, cognitive, expressive-motor, and behavioral responses and can be adaptive when they help the body process information which is quick and automatically gathered during an event [39]. A cyberbullying incident can generate different emotions in victims, aggressors, and bystanders [[41], [42], [43], [44], [45], [46]]. Cyberbullying has been associated with unpleasant emotions, especially in victims, and pleasant emotions [3]. Some of these emotions includes those referred by Ekman [38]. Although the aim of this investigation is to study the emotions felt by bystanders in different scenarios of cyberbullying, to better understand emotions, it is also important to mention some emotions felt by victims and aggressors. Thus, according to the literature, victims tend to feel sadness, anger, fear, shame, depressive symptoms and anxiety [[41], [42], [47], [48]], whereas the most prevalent emotion in aggressors is anger [49].

With regard to the emotions experienced by bystanders, Caravita et al. [49] mentioned that girls tended to experience more anxiety than boys, which may be related to their greater tendency to internalize symptoms and to use more emotion-focused coping strategies [50], resorting to rumination, whereas boys are better able to distance themselves from the situation [49,51,52].

### 1.3 Emotion regulation in cyberbullying

Several theoretical models can be considered when examining emotion regulation in the context of cyberbullying. For instance, the Social Cognitive Theory [53] has suggested that individuals learn behavior through observation. It also emphasizes the dynamic interplay between individual, contextual and behavioral factors which influence human behavior. Therefore, in the context of cyberbullying, this theory can provide insights to understand how adolescents learn and adopt emotion regulation strategies by observing others within specific contexts, but that their own individual characteristics are important in determining how they react. Gross [54] defined emotion regulation as the use of conscious or unconscious strategies which are used to increase, maintain, or decrease the various components of emotional response (i.e., behavior, cognitions and physiological responses). As mentioned above, emotion regulation is an important predictor of psychological adjustment and social competence in adolescents [12,21,55] and according to the literature [[21], [56], [57]], one of the causes of cyberbullying is the difficulty revealed by aggressors in regulating their emotions adaptively. In fact, the higher the emotional regulation, the lower the prevalence of cyberbullying. On the contrary, the lower the emotional regulation, the higher the cyberbullying prevalence [12,58]. Specifically, individuals who can regulate their emotions seem to reveal a greater capacity to control impulsive behavior, obtain more emotional support and establish better interpersonal relationships [59]. However, lower emotion regulation indicates a subsequent aggressiveness in relationships, which has a negative impact on the quality and quantity of interpersonal relationships, resulting in more loneliness experienced by adolescents [60,61]. This perspective complements the Transactional Model of Stress and Coping [50], which proposes that emotion regulation can be considered as a coping mechanism, which influences how individuals react to and cope with difficult emotional experiences. Thus, cyberbullying is experienced as a stressful event to which individuals react to and must cope with.

For victims' strategies of action to be the most appropriate and for bystanders to adopt pro-social behavior when facing cyberbullying events, it is important that those involved can regulate their emotions adaptively [21]. In line with this, some studies [62] have correlated maladaptive strategies to self-blame (thoughts of blaming oneself for what is being experienced), blaming others (focusing the blame for what has been experienced on others), rumination (constantly thinking about the feelings and thoughts generated by the situation) and catastrophizing (having recurring thoughts about the seriousness of the event, emphasizing terror and the fact that it is the worst experience that can happen). On the other hand, adaptive strategies include acceptance (the act of recognizing what has happened and accepting it, often involving a feeling of resignation to the reality of the situation), refocusing on planning (thinking about the next steps to take and how to manage the negative event), positive refocusing (focusing on happy and pleasant issues or positive experiences, instead of thinking about the current situation), positive reappraisal (assigning a positive meaning to the negative situation in terms of personal growth) and perspective (minimizing the importance of the negative event, highlighting its relativity when compared to other situations). The Affect Regulation Theory [63] complements this approach by highlighting the role of emotion regulation to maintain individuals’ emotional state. It proposes that individuals use different strategies to regulate their emotions and maintain emotional well-being. Moreover, this theory can be useful in understanding emotion regulation in cyberbullying situations. Specifically because it underscores how emotion regulation strategies help manage the emotional impact of cyberbullying.

In the present study, we adopted the Process Model of Emotion Regulation [39] to study how adolescents regulate their emotions in different cyberbullying situations considering the five main categories of specific emotion regulation strategies, which can be used to modify the course of emotions, which are: (1) Situation selection (approaching or avoiding certain situations or objects based on the psychological impact that is expected to occur); (2) Situation modification (modifying physical aspects of the situation or environment); (3) Attentional focus modification (choosing where to focus attention, whether on non-emotional situations (distraction) or on negative emotional situations in a repetitive way (rumination); (4) Cognitive modification (changing the meaning given to the evaluation of the situation through cognitive reappraisal, for example); (5) Response modulation (modifying the expressive, behavioral, or physiological response components of the emotion, through suppression (inhibiting facial expression), biofeedback (muscle relaxation) and acceptance (acceptance of the emotion and its consequences) [39,[64], [65], [66], [67], [68], [69], [70], [71]].

Many authors have investigated the regulation strategies adopted by adults, but few studies exist on the emotion regulation strategies adopted by adolescents (e.g., Ref. [22,72]), calling for more studies in this area, since it is during adolescence that the help provided by caregivers to maintain adaptive emotion regulation in different contexts decreases [72,73]. Therefore, the likelihood of youth adopting inappropriate behavior at this stage of development is greater, given that they are more prone to be involved in impulsive and risk behavior, particularly when there is a greater usage of social media [74].

Additionally, researchers have stated that more studies are needed to explore emotion regulation in different contexts and to explore differences in sex which is in line with the present research [75,76]. Thus, it is crucial to understand the emotional reactions of bystanders in these incidents, as well as the emotion regulation strategies [39] they adopt to deal with the phenomenon and intervene in a pro-social manner. This study seeks to describe the emotions and emotion regulation strategies that adolescent bystanders adopt in different types of cyberbullying aggression, since it is recommended to develop intervention and prevention programs for cyberbullying in schools focusing on emotion regulation [21,77,78], which reinforces the relevance of this study.

### 1.4 Aims of the study and research questions

The current study presents three main objectives. Firstly, we proposed to analyze the emotions and emotion regulation strategies adopted by adolescents after observing various cyberbullying behavior. Secondly, we intended to investigate the influence of the variable sex on the emotions felt by cyberbullying bystanders and the emotion regulation strategies after they observed four different cyberbullying scenarios. Thirdly, we aimed to investigate the role of the school grade on bystanders’ emotions and emotion regulation strategies after observing these scenarios. According to these objectives, we propose the following research questions.

• What are the emotions and emotion regulation strategies adopted by bystanders of cyberbullying in different cyberbullying scenarios?
• Are there differences between boys and girls in terms of the emotions felt and emotion regulation strategies adopted when facing different cyberbullying scenarios?
• Are there differences between 7th grade and 8th grade students in terms of the emotions felt and emotion regulation strategies adopted when facing different cyberbullying scenarios?

## 2 Methodology

### 2.1 Ethics statement and design

Data is confidential due to the nature of the study and was an obligatory requirement of the Faculty of Psychology of the University of Lisbon's Ethics Committee, whereas material is available upon request to the authors. This study was approved by the Ethics Committee of Faculty of Psychology of the University of Lisbon, with ethics approval reference 5417/2016.

For a better understanding of the research problem, a mixed methods approach was adopted, defined by the combination of quantitative and qualitative data [79]. This study used a quasi-experimental design with pre and post-test measures, which was complemented by a qualitative approach with individual interviews. The study used qualitative and quantitative methodologies to explore adolescents' responses to cyberbullying. The qualitative aspect focused on exploring emotions and emotion regulation strategies through individual interviews, providing an in-depth understanding of bystanders' experiences. Concurrently, this study employed quantitative methods to analyze the impact of variables such as sex and school grade on emotional reactions. Surveys with structured questions facilitated statistical assessments, allowing for a comprehensive exploration of how specific factors have an impact on emotional and regulatory dynamics among cyberbullying bystanders. The integration of both approaches ensures a thorough examination of adolescents' emotional responses in cyberbullying scenarios.

### 2.2 Participants

In this study, 140 adolescents took part in a convenience sample attending a school in the district of Lisbon, considering their availability to take part in the study and because it is a school in the country's capital where there are many students from different social and cultural backgrounds, although, cultural issues or those related to socio-economic status were not considered in this study.

From the total sample, 68 of the participants were in the 7th grade (48.2 %) and 72 were in the 8th grade (51.8 %) and 51.1 % were female. The ages ranged from 12 to 17 years old, with the average age being 13 years old. The study consisted of a face validity, a longitudinal study with five sessions with the participants playing a serious game, Com@Viver and a final qualitative study with individual interviews. The face validity was conducted with 28 students that did not participate in the rest of the study. As for the longitudinal study, classes were randomly assigned to either the control group or the experimental group.

There were 91 students in the control group condition divided into two groups. Control Group 1 (GC1) did not play the serious game on the computer but had an alternative intervention. GC1 had access to the content of the same game in paper format, i.e., with the same posts and possibility to react to the posts, and they could also choose pre-defined comments to react to the posts and pre-defined messages in the chat. In Control Group 2 (CG2), they interacted through a fictitious social network on topics related to organizing a field trip. In the meantime, real cyberbullying situations appeared, and participants could react to them in the same way as CG1, in the computer. At the end of the session after students of CG1 and CG2 had reacted to their actions in the network, a researcher in the room would check the answers given by the students and attributed a score using the same process as in the experimental condition, after playing the serious game Com@Viver.

The 101 students in the experimental group played Com@Viver in the schools’ computers. Six classes participated, three of which were 7th graders and the other three 8th graders. In a second phase, from this sample, 26 students randomly volunteered to participate in an individual interview, and of these, 61.5 % were female. The study started on the March 9, 2018 and ended on the June 8, 2018.

### 2.3 Instruments

#### 2.3.1 Emotions in cyberbullying in adolescents questionnaire and the emotion regulation of adolescents in cyberbullying questionnaire

The quantitative approach of this study involved the use of two online questionnaires embedded in the Qualtrics XM Online platform. To identify emotions and emotion regulation strategies in adolescents, the Emotions in Cyberbullying in Adolescents questionnaire (ECA) and the Emotion Regulation of Adolescents in Cyberbullying questionnaire (ERAC) were used. The ECA was adapted from the Inventory of Observed Cyberbullying Incidents (IOCI, [80]), which aims to analyze the behavior of bystanders in cyberbullying incidents. Among the various scales that are included the IOCI, the ECA measures 20 emotions. The literature has shown that in cyberbullying incidents there are certain emotions that are more reported by bystanders than others [21] and, for this reason, the ECA was built based on the existing literature and on the results obtained by the IOCI [41,42,81].

We computed exploratory factor analysis to interpret the internal structure of the instruments. We verified multivariate normality with Mardia's coefficient, which should be lower than P(P + 2). P is the number of observed variables. Therefore, 20 observed variables in the emotion instrument questionnaire showed a Mardia's coefficient for skewness of 215.572 < 18(18 + 2) = 440 and for kurtosis of 846.59 > 8(8 + 2) = 440 [82]. Due to kurtosis, we chose Robust Unweighted Least Squares (RULS) to extract factors, as it does not depend on distributional assumptions (Joreskog, 1977). The Kaiser-Meyer-Olkin measure of sampling adequacy was 0.94 and Bartlett Sphericity was χ^2^_28_ = 5637.9 (*p* < 00.001), revealing that the variables were adequate for factor analyses. Two items were removed because of factor loadings below 0.30. We used Optimal implementation of Parallel analyses to determine the appropriate number of factors to retain [83]. Two factors were obtained with 64 % of explained variance for emotions (pleasant and unpleasant). Both factors presented good reliability scores (i.e., pleasant emotions = 0.88; unpleasant emotions = 0.97 [84]. The inter-factor correlation was 0.72. All remaining 18 items revealed good loadings (from 0.47 to 0.99) and were significantly correlated (See Appendix A, Table A1).

We performed confirmatory factor analysis with IBM, SPSS AMOS v. 25 with estimation procedures of unweighted least squares and checked the fit indices such as chi-square, Standardized Root Mean Square Residual (SRMR), Goodness of Fit Index (GFI) and Normed Fit Index (NFI). The GFI and NFI values close to one indicate a good statistical fit [85], whereas SRMR indicates a good fit if close to zero [86]. CFA values included χ2 (130) = 522.69, p < 00.00, GFI = 0.99, NFI = 0.99, RFI = 0.99, SRMR = 0.06. The analysis confirmed a two-factor model with pleasant (i.e., “I felt happy”. “I felt indifferent.” “I felt secure.” “I felt proud.” “I felt strong.” “I felt enthusiastic.”) and unpleasant emotions (“I felt sad.”; “I felt revolted.”; “I felt avenged.”; “I felt defenseless.”; “I was afraid.”; “I was enraged.”; “I felt angry.”; “I felt anguished.”; “I felt threatened.”; “I felt irritated.”; “I felt embarrassed.”; “I felt worried.”) with factor scores between 0.48 and 1.

Although there is an emotion regulation questionnaire for children and adolescents in the literature (The Emotion Regulation Questionnaire for Children and Adolescents - QRE-CA; [87]) that includes two of the five emotion regulation strategies of Gross model [39] (i.e. cognitive reappraisal and emotional suppression), this questionnaire is not directed to online contexts nor to cyberbullying situations. For this reason, it was deemed important to construct a questionnaire that explored all the emotion regulation strategies of the model [39] adapted to the online context in cyberbullying situations [22,70,72]. The ERAC was subjected to exploratory analysis and confirmatory analysis. ERAC was initially created with 22 items and after Factor Analysis v.12.02.01 8 items were removed (item 1 – “I didn't feel anything because I didn't notice”, item 3 – “I didn't pay attention to the situation to not get into trouble”, item 6 – “I skipped the posts to avoid being bothered”, item 7 – “I looked at other posts that made me feel differently”, item 8 – “I recalled situations that made me feel different”, item 21 – “I continued what i was doing to ignore the situation”, item 21 “I continued what I was doing to ignore the situation” and item 22 – “I showed what I felt to make my point”. It showed a Kaiser-Meyer-Olkin KMO = 0.910, Bartlett Statistic = 9074, with degrees of freedom 77. One dimension resulted from the factor analysis, namely, the “Youth Emotion Regulation Strategies to Cope with Cyberbullying” (*α* = 0.93) consisting of 14 items. The adolescents' answers were recorded on a 4-point Likert-type scale (1 – “*Not at all*” to 4 – V*ery much*).

#### 2.3.2 Semi-structured interview

**Fig. B.1:**
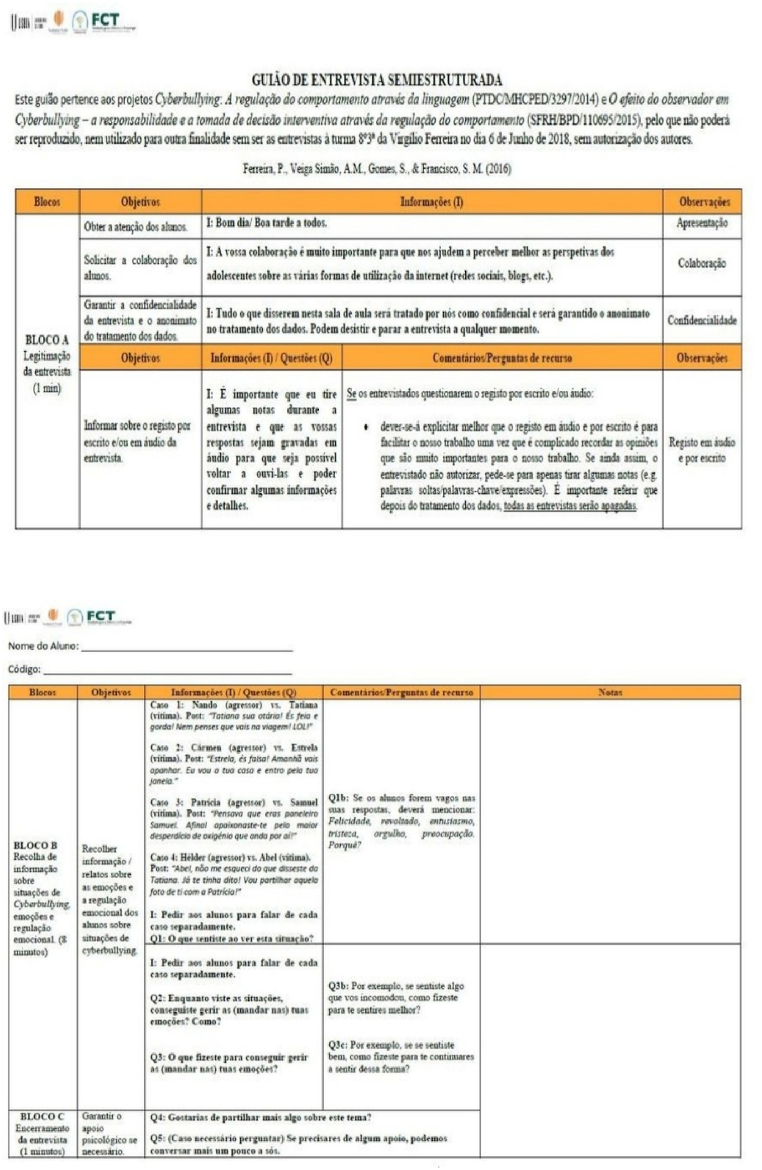
Semi-structured interview script

The qualitative approach involved a 20-min, audio-recorded, semi-structured individual interview with seven questions directed toward the emotions felt in each observed cyberbullying scenario and the emotion regulation strategies adopted to cope with those emotions (See Appendix B, Figure B1). This interview aimed to collect information/reports about students' emotions and emotional regulation strategies regarding cyberbullying situations they observed. The interview was divided into three blocks named A, B and C. The first (block A) aimed to ensure the confidentiality and the anonymity of the interview and data processing. The students were also informed about the importance of writing and audio recording their answers and we requested their authorization to do so (e.g., “*It is important that I take some notes during the interview and record your answers, so that I can listen to them again and be able to confirm some information and details*.”). Block B aimed to collect reports about the emotions and emotional regulation strategies in cyberbullying situations (e.g., “*If you felt something that bothered you, how did you make yourself feel better?*”). Finally Block C concluded the interview (e.g., *“Is there anything else you would like to share about this topic?”*). Specifically, students were asked about what emotions they felt in each observed scenario (e.g., “What did you feel when you saw this situation?”). In addition, if the reported emotions were unpleasant, the students were asked about what they did to feel better (e.g., “For example, if you felt something that bothered you, how did you make yourself feel better?”). If the emotions they felt were pleasant, they were asked to indicate what they did to continue feeling that way (e.g., “For example, if you felt good, what did you do to continue feeling that way?”).

### 2.4 Educational resources – the game Com@Viver

Com@Viver is a serious digital game that is part of a research project (“The Bystander Effect in Cyberbullying: taking responsibility and intervening through the regulation of behavior in adolescence - SFRH/BPD/110695/2015) that aims to investigate bystander behavior in cyberbullying incidents and to promote prosocial behavior in this context.) “Serious games are based more on the objective than on the games themselves” [88] and aims to create an impact on the players' lives in a meaningful way by promoting behavior change. There are few studies (e.g., Ref. [89]) assessing validated games’ efficacy in real world settings [90], and no studies using these resources to intervene in a longitudinal manner with social interaction among peers [91,92]. Thus, C@mViver game was created based on the Social Cognitive Theory [93] and the Bystander Intervention Model [94] and we believe that by playing a game that recreates a real social network, participants become more involved, reducing bias.

The goal of the game is to organize a field trip, although there are a limited number of seats available on the bus for the trip. It is a multiplayer game where players form groups of three students from the same class who can interact with each other but cannot interact with the other groups of three players. The interactions occur through a fictional social network with 12 social agents who interact with each other and the players [91,92]. The posts that appear in the news feed of this social network are essentially centered on the organization of the trip and everyday situations. This game was applied over five weekly sessions (from session 0 to session 4). Session 0 is a practice trial session. From session 1 of the game, the social agents begin to adopt aggressive behavior that recreates hypothetical cyberbullying situations to which adolescents can react to by clicking “like” or “dislike”. They can also comment on the post by selecting from the predefined options which include supporting the victim, supporting the aggressor, or not reacting.

In the four cyberbullying situations presented (from sessions 1 to 4) in the game, there is also a Chat, in which the players can select one or more answers, which are also predefined, to comment on the situations they observed. These answers are not visible to the other players on the team. Briefly describing the scenarios, in the 1st scenario the aggressor publishes a photograph of a girl in a bikini with the intention to denigrate her image, make fun of her and exclude her; whereas in the 2nd scenario the aggressor publishes a direct threat to the victim; in the 3rd the aggressor insults the victim through a publication written in the Com@Viver feed; and in the 4th scenario the aggressor threatens to share the victim's personal information, without her permission, to spread rumors.

To minimize some confounding variables that may have influenced the study, some measures were taken. For example, during the game, all participants used headphones to avoid distractions. So that the students wouldn't forget the codes of the group to which they belonged, the researchers distributed them to the students and checked that they had entered the correct codes at the beginning of the session. The teacher who taught the course was always present during the session, as well as a master's student from the computer engineering course at the University of Lisbon to correct any technical difficulties that might occur.

### 2.5 Procedures

**Fig. C.1:**
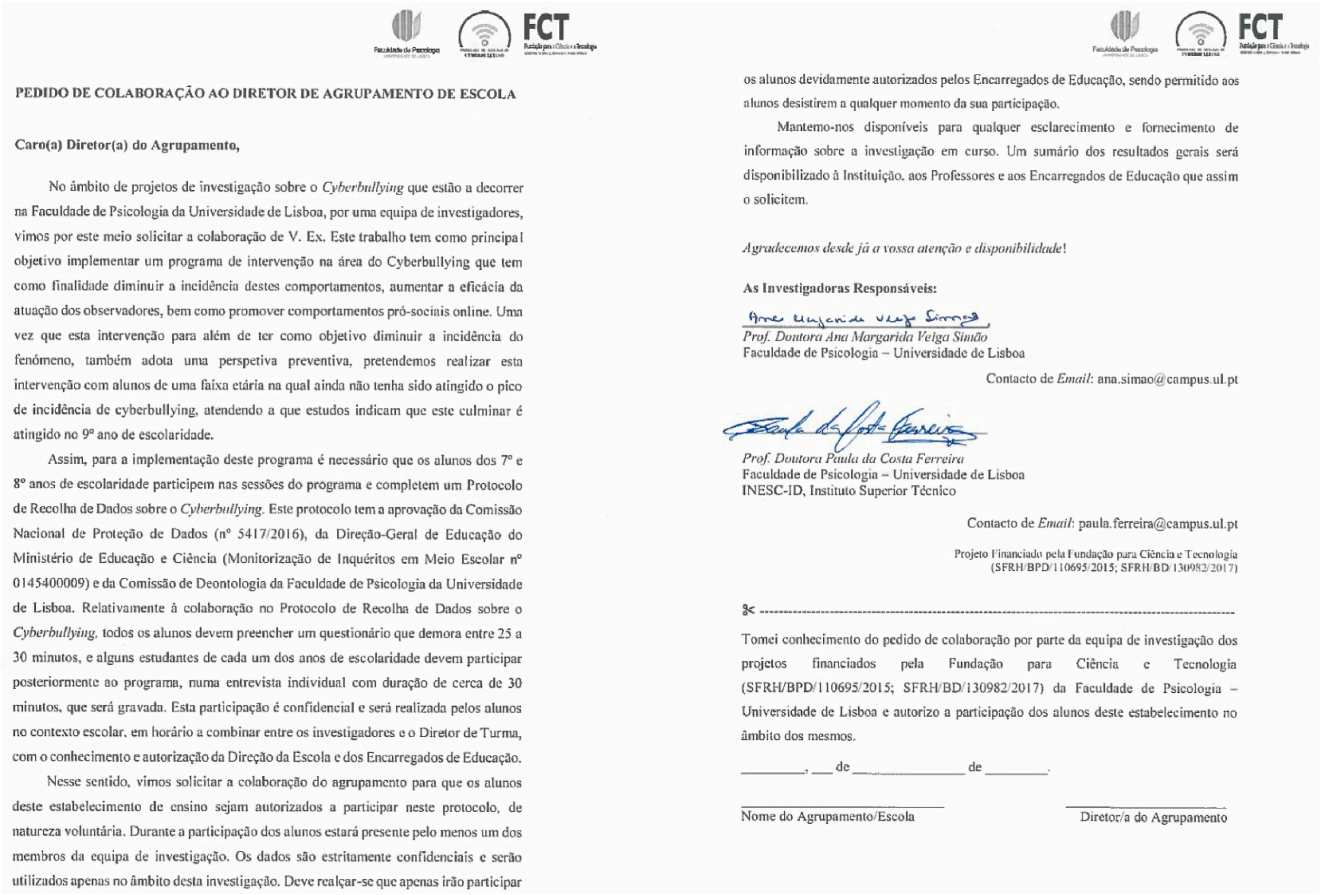
Authorization request to the Director

**Fig. C.2:**
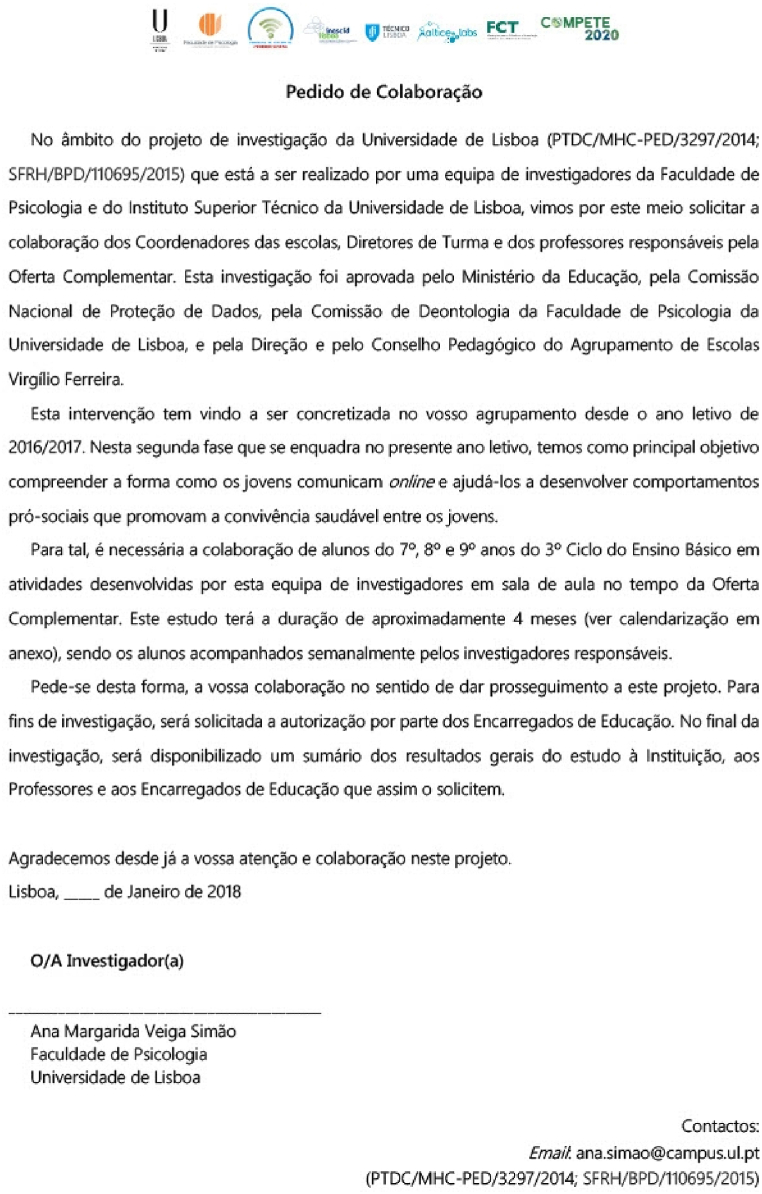
Authorization request to Teachers

**Fig. C.3:**
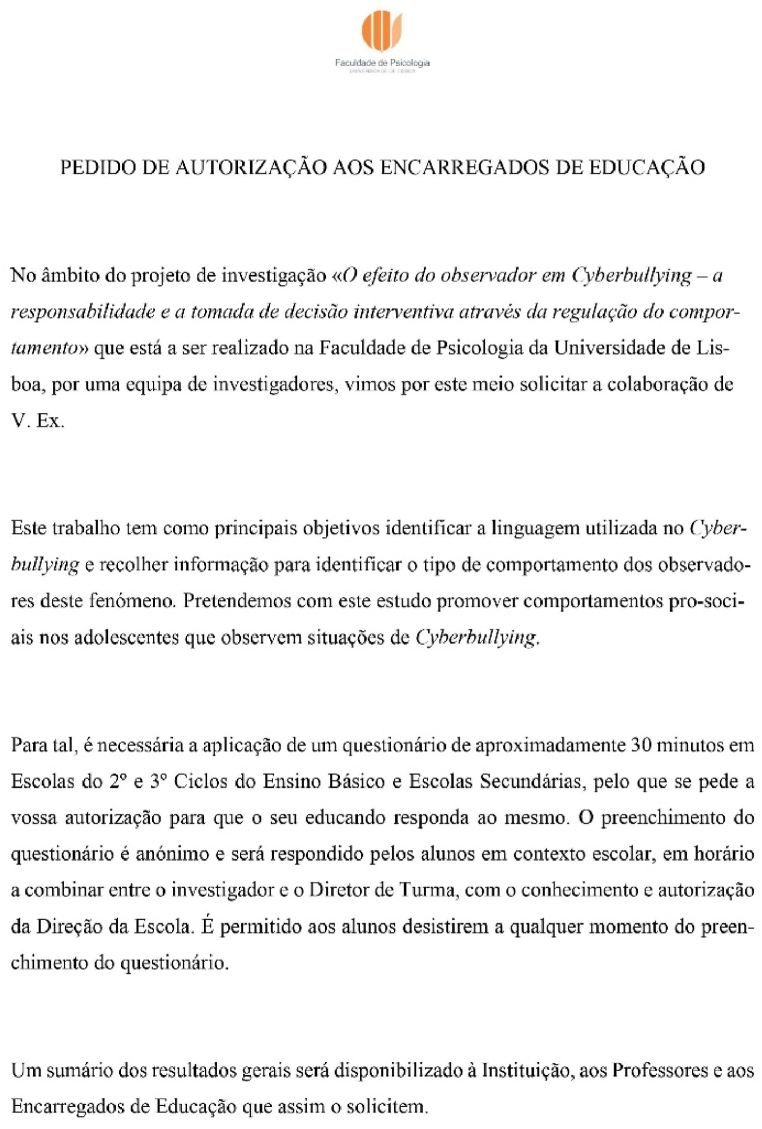
Authorization request to Parents

**Fig. C.4:**
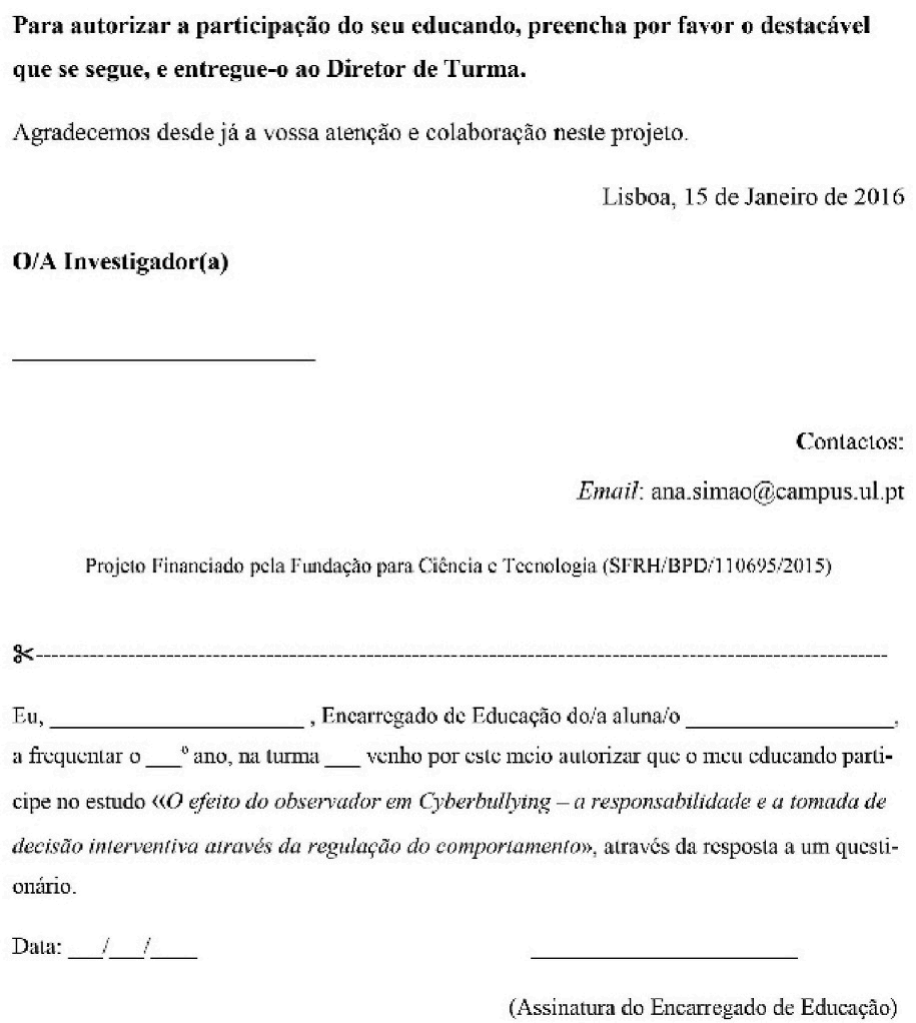
Information given to participants about the study and informed consent

This research includes experiments with human participants, so authorizations were requested and collected from the Portuguese Ministry of Education, the Portuguese National Commission for Data Protection, the Deontology Committee of the Faculty of Psychology of the University of Lisbon, with approval and authorization number 5417/2016, the Director of a public-school cluster in the Lisbon region (See Appendix C, Figure C1) the teachers (See Appendix C, Figure C2), the parents (See Appendix C, Figure C3) and the adolescents themselves (See Appendix C, Figure C4). All participants were informed of the possibility of requesting psychological support (i.e., with a clinical psychologist) if they needed to talk to someone while completing the questionnaires. Furthermore, all students were informed of the possibility of withdrawing from completing the questionnaires or even from participating in this study at any time. Researchers administered the questionnaires, applied the educational resources (e.g., game Com@Viver) and conducted the interviews.

Before the intervention, students conducted pre-tests by filling out the questionnaires individually on a computer (experimental group) or in paper format (control groups). Then, the students played the game (without the cyberbullying cases) on the computer (experimental groups) to know how the game functioned, with the researchers’ supervision. After this moment, the students played the game weekly on the computer or in paper format (experimental and GC1, respectively), where during each week, a different cyberbullying situation arose. At the end of each session, the students filled out the ECA where they indicated the emotions they felt with regard to the cyberbullying situation presented.

**Fig. 1:**
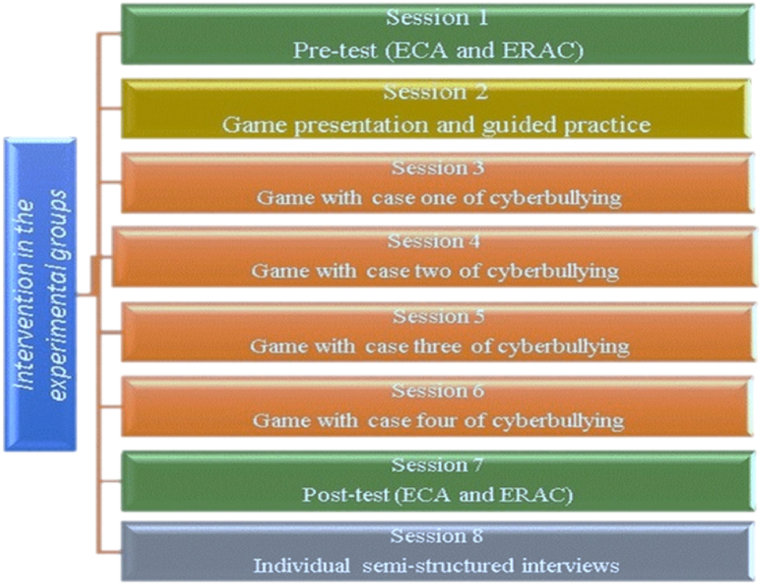
Conceptualization of the experimental intervention.

At the end of the four weeks, students conducted the post-test. Lastly, 26 students from the experimental group volunteered to participate in the semi-structured interviews focusing on how they felt, and the emotion regulation strategies which they used to deal with the respective emotions felt in each scenario (see Fig. 1).

**Fig. 2:**
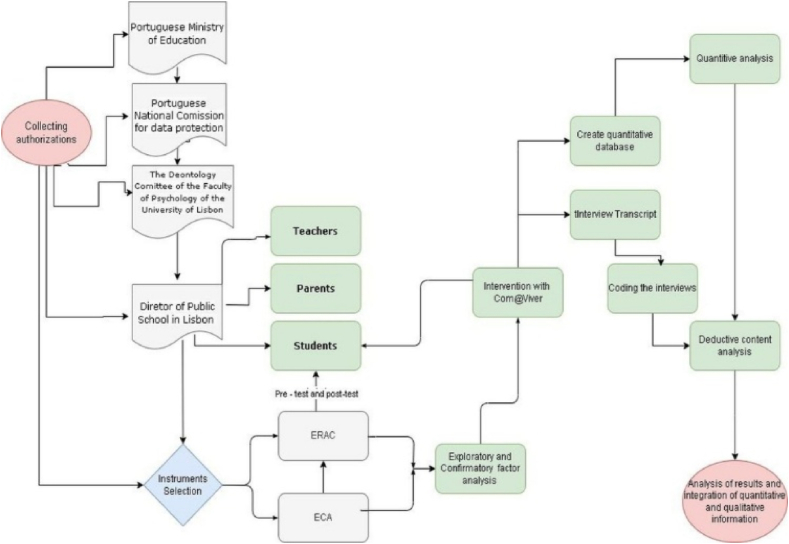
Research flowchart.

The interview was conducted to further investigate the variables under study, without the interference of the peer group. The individual interviews were conducted by researchers in a classroom available for the purpose of ensuring the participants’ privacy. In this research, participants always adopted the role of bystanders. The interviews were audio-recorded, after obtaining permission from the parents and the students themselves to allow for data transcription. Once the interviews were completed, they were transcribed into a word document, and it was given a number to each participant. Then, a deductive content analysis was carried out in which the emotions reported were categorized into those that are part of the ECA. The same happened with the emotional regulation strategies, which were categorized into the different strategies of James Gross emotional regulation. Fig. 2 shows a flowchart of the procedures adopted.

### 2.6 Data analysis

This study uses exploratory, descriptive and correlational analysis. To this end, quantitative data collected from the ECA and ERAC were entered into the IBM SPSS 22 computer software. The quantitative data were subjected to a frequency analysis of the students’ answers, as well as the averages of the answers given for the variables under study. In addition, the effect of the variables sex (based on biological differences) and year of schooling on the variables emotions and emotion regulation strategies for the four scenarios was investigated using the nonparametric Mann-Whitney test, once the population data does not have a normal distribution and these variables (sex and year of schooling) are independent groups.

The qualitative data collected in the individual interviews was transcribed into a word document and it was given a number to each participant. Each participant was assigned a unique identifier or number for anonymity and organizational purposes. A deductive content analysis approach was employed, aligning with a pre-existing framework (ECA). This framework provided predefined categories and subcategories to guide the analysis of the qualitative information. The same occurred with the emotional regulation strategies, which were categorized into the different strategies of James Gross' [39] emotional regulation model. This step involved counting the occurrences of specific themes or content related to emotional reactions and regulation strategies. Emotional regulation strategies were further analyzed using James Gross' [39] established model. The qualitative information was categorized into the specific strategies outlined in Gross’ model, providing a structured framework for understanding how participants regulated their emotions in response to cyberbullying scenarios. To ensure rigor, the coded data and interpretations were subjected to a thorough review by multiple researchers involved in the study. This collaborative process aimed to enhance the reliability and validity of the findings. Researchers engaged in reflexive practices, acknowledging, and documenting their own biases and perspectives throughout the coding and interpretation process. Consistency in coding decisions was maintained through regular meetings and discussions among the research team.

## 3 Results

### 3.1 A quantitative analysis of the emotions and emotion regulation strategies adopted by bystanders in cyberbullying

In Table 1 we present the means and standard deviations of the quantitative variable emotion, for the total sample, concerning the different cyberbullying scenarios. In the four scenarios, most participants reported feeling unpleasant emotions, when they observed the different cyberbullying situations. Out of the four scenarios, in the fourth scenario, participants reported feeling slightly less unpleasant emotions than in the remaining scenarios.

**Table 1:** Means and standard deviations of emotions felt by bystanders.

Variables	Scenario 1		Scenario 2		Scenario 3		Scenario 4	
	Mean	SD	Mean	SD	Mean	SD	Mean	SD
*Pleasant emotions*	1.20	0.47	1.20	0.50	1.20	0.50	1.20	0.49
*Unpleasant Emotions*	2.90	0.89	2.90	0.95	2.90	0.97	2.80	1.03

Table 2 presents the means and standard deviations regarding emotion regulation strategies. The results revealed that most of the participants sought to interpret what they were feeling to try to help more in scenario 1 than in the other scenarios. The emotion regulation strategies used to deal with their emotions revealed no differences between the four scenarios.

**Table 2:** Means and standard deviations of emotion regulation strategies.

Variables	Scenario 1		Scenario 2		Scenario 3		Scenario 4	
	Mean	SD	Mean	SD	Mean	SD	Mean	SD
*Interpretation of the emotional experience*	2.40	0.81	2.30	0.85	2.30	0.88	2.20	0.87
*Emotion regulation strategy to deal with emotional experience*	1.70	0.57	1.70	0.65	1.70	0.69	1.70	0.68

The most commonly used emotion regulation strategies in the four scenarios (see Table 3) were approaching the situation to try to help, with an equally high value in scenario 1 (79 %) and scenario 2 (74 %) and a lower value in scenario 4 (62 %). We verified that adolescents had the ability to put themselves in the victim's place to understand their feelings, with a higher value in scenario 1 (74 %) and scenario 3 (72 %). This last strategy was the most used in the scenarios referring to the publication of photos of others or the publication of homophobic comments directed at others. Of the total sample, 108 (77 %) of the participants put themselves in the victim's shoes in scenario 1, 107 (76 %) of adolescents in scenario 2, and 106 (75 %) did as well. The aggressive behavior that involved publishing others' photographs, insulting others or the behavior that involved spreading rumors about others' personal lives triggered concern for the victim in scenario 4. Of the four scenarios, the one that had the greatest impact on adolescents, in terms of them realizing what was happening, putting themselves in the victim's shoes to understand what she was feeling to try to help and changing the situation, was scenario 1, which involved the publication of another's photograph.

**Table 3:** Frequencies and percentages of emotion regulation strategies for interpreting emotional experience.

Variables	Scenario 1		Scenario 2		Scenario 3		Scenario 4	
	N	%	N	%	N	%	N	%
**Situation selection –** **Approach**	111	79	105	74	94	67	88	62
**Modifying attention focusing – rumination**								
*About what was seen*	94	67	85	60	87	62	82	58
*About what was felt*	91	65	85	60	86	61	89	63
*About what peers felt*	83	59	85	60	77	55	88	62
*About what the cyberbully felt*	87	62	82	58	84	60	80	57
*About what the victim felt*	104	74	99	70	101	72	94	67
*About a similar situation*	73	52	68	48	64	45	65	46
**Cognitive restructuring**								
*Taking the cyberbully's perspective*	85	60	76	54	74	53	79	56
*Taking the victim's perspective*	108	77	107	76	97	69	106	75

As for the description of the emotion regulation strategies most used to deal with the emotions felt by adolescents in the different scenarios (see Table 4), the results showed that it was in scenario 1 (36 %) and scenario 3 (32 %) that they mostly kept to themselves what they were feeling (51 %) through emotional suppression. Also, in these two scenarios (32 % and 28 % respectively), students used cognitive change by altering the way they viewed the situation. For the total sample, the emotion regulation strategy of distraction was the most used in scenarios 1 and 3 (32 % and 27 %, respectively) and rumination was the most used in scenarios 1 and 2 (32 % and 26 %, respectively). In summary, the scenarios referring to aggressive behavior involving the publication of photos of others and the publication of homophobic comments triggered adolescents to use the emotion regulation strategies of distraction, cognitive change, and emotional suppression to cope with the emotional experience more often.

**Table 4:** Frequencies and percentages of emotion regulation strategies for coping with emotion.

Variables	Scenario 1		Scenario 2		Scenario 3		Scenario 4	
	N	%	N	%	N	%	N	%
**Situation Selection – avoidance**	29	21	25	18	26	18	22	16
**Modifying attention focusing – distraction**	45	32	37	26	39	27	35	25
**Modifying attention focusing – rumination**	45	32	37	26	33	26	35	25
**Cognitive Restructuring**	45	32	36	25	40	28	37	26
**Emotional Suppression**	51	36	40	28	45	32	40	29
**Emotional Suppression – biofeedback**	16	11	24	15	23	16	25	18

### 3.2 A qualitative analysis for the emotions and emotion regulation strategies adopted by bystanders in cyberbullying

**Fig. 3:**
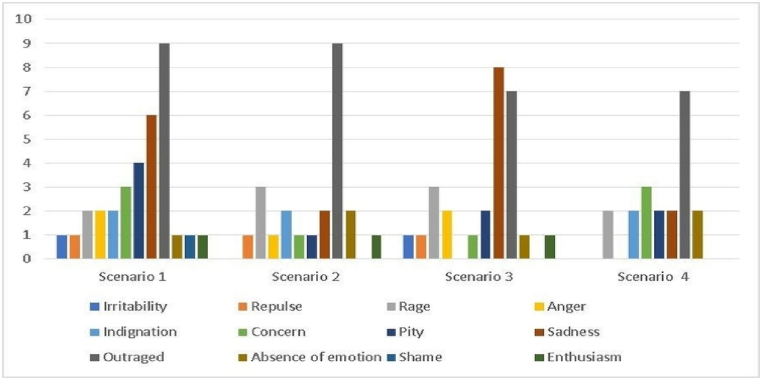
Emotions felt by the adolescents in the four different scenarios.

The qualitative results indicated that the emotion most reposted by adolescents in the four scenarios was anger (most frequently in scenarios 1 and 2), which refers to the publication of a photograph of other people without their permission and to threats. Scenario 1 was the one that most aroused compassion for the victim. Scenarios 1 and 4 caused adolescents the most concern, and scenarios 1 and 3 caused the adolescents the most sadness. The publication of photographs and insults generated more unpleasant emotions in adolescents than the other scenarios (see Fig. 3). For example, participant 7 (P7) said: *“unfair and angry” and participant 1 (P1) said, “I think this was the worst of all, Horrible.”*

**Fig. 4:**
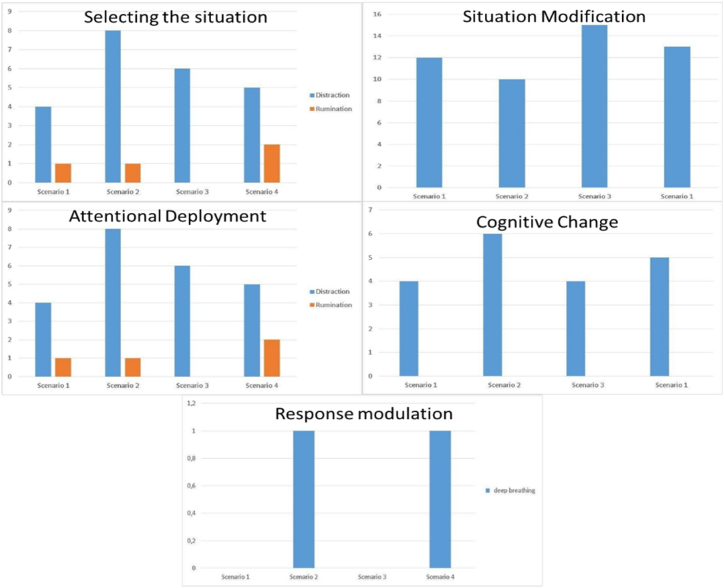
All emotion regulation strategies used by adolescents in different cyberbullying scenarios.

Faced with the emotions felt by adolescents, the emotion regulation strategies used by them varied (see Fig. 4).

Concerning the strategy of selecting the situation, scenario 1 generated more situations of avoidance in adolescents in dealing with unpleasant emotions. For example, participant 13 (P13) said “*Not talking about it*”, and in scenarios 1 and 3 most of the adolescents tried to understand what was happening to be able to help. Regarding the strategy of changing the situation, scenario 1 was the situation where adolescents were most mobilized to change the situation (P3: “*Reporting or telling others that it was wrong*”), followed by scenario 3 (P9: “I commented in the chat that it was wrong”).

Scenario 2 was where there was a higher frequency of emotion regulation strategies involving distraction, since some adolescents reported being used to seeing these situations on social networks and in everyday life (P2, *“I didn't feel any emotion, it's something I'm used to seeing*”; P9, “*I didn't pay much attention*”). However, other participants mentioned that what they were seeing bothered them, and therefore, they moved on to other more entertaining posts (P13, “*I saw other, livelier posts*”). Scenario 1 was the one in which adolescents used the distraction strategy the least because they considered it a more serious situation and sought to do something to help (P25: “*concerned about the victim”).* The strategy of cognitive change was the most used in scenario 2, with adolescents considering the situation less serious because they believed it was not a real threat (P10: “*funny, no one threatens someone like that online*,”; P6: “*I thought it was a lie*”). In threatening situations of sharing the victim's personal information (Scenario 4), one participant reported using the strategy of taking a deep breath to calm down (P3: “Tried to take a deep breath). In the remaining scenarios, this strategy was not used.

In short, most adolescents used emotion regulation strategies to try to resolve the situation, as previously verified in the quantitative data collected. Scenario 1 seemed to be the one that had the most impact on adolescents. In addition, some adolescents mentioned that the scenarios involving threats generated emotions of indifference because it was something they were used to seeing in their daily lives (P3: “*Indifference, we are used to see it”*.

### 3.3 Differences between boys and girls in terms of emotions and emotion regulation in cyberbullying

To test the relationship between sex and the variables pleasant and unpleasant emotions and emotion regulation strategies, interpretation of emotional experience and management of emotional experience in the four scenarios, the data was analyzed using the Mann-Whitney test. The results are shown in Table 5 (for sex, emotions and regulation strategies*)* and Table 6 (for sex and strategies for interpreting emotional experience) below. This test showed that the sex variable had an effect on negative emotions in scenarios 2 (*U* = 1774.500; *P* < 0.005), 3 (*U* = 1719.000; *P* < 0.035) and 4 (*U* = 1938; *P* < 0.05), but no effect in scenario 1 (*U* = 2115.000; P > 0.05). This means that there were no differences between the negative emotions felt by girls and boys in scenario 1. Scenario 1 triggered unpleasant emotions in both boys and girls. However, in scenarios 2, 3 and 4, girls showed higher responses than boys for negative emotions. For the emotion regulation strategy variables, the test showed that sex had an effect on the interpretation of emotional experience in scenarios 2 (*U* = 1844.000; *P* < 0.05), 3 (*U* = 1894.500; *P* < 0.05) and 4 (*U* = 1930.500; *P* < 0.05) and no effect on scenario 1 (*U* = 2023.500; *P* > 0.05). This means that in scenario 1, both boys and girls sought to interpret the emotion of what they were feeling to be able to help. Table 6 indicates the emotion regulation strategies for the variable interpretation of an emotional experience where there were differences between boys and girls, in the four scenarios. The results showed that the sex variable had an effect on the emotion regulation strategies “situation selection - approach” (U = 1947.000; P < 0.05) and the variable “situation modification” (U = 1770.500; P < 0.05).

**Table 5:** Mann Whitney test results for sex, emotions, and regulation strategies.

Variables	Sex	N	U	*P*
Pleasant emotions scenario 1	F	71	2613.00	0.32
	M	69		
Unpleasant emotions scenario 1	F	71	2115.00	0.15
	M	69		
Pleasant emotions scenario 2	F	71	2667.50	0.12
	M	69		
Unpleasant emotions scenario 2	F	71	1774.50	0.00^a^
	M	69		
Pleasant emotions scenario 3	F	71	2667.50	1.13
	M	69		
Unpleasant emotions scenario 3	F	71	1719.00	0.03^a^
	M	69		
Pleasant emotions scenario 4	F	71	2663.50	0.12
	M	69		
Unpleasant emotions scenario 4	F	71	1938.00	0.03^a^
	M	69		
Interpreting emotion scenario 1	F	71	2023.50	0.07^a^
	M	69		
Dealing with emotion scenario 1	F	71	2495.00	0.84
	M	69		
Interpreting emotion scenario 2	F	71	1844.00	0.01^a^
	M	69		
Dealing with emotion scenario 2	F	71	2414.00	1.00
	M	69		
Interpreting emotion scenario 3	F	71	1894.50	0.02^a^
	M	69		
Dealing with emotion scenario 3	F	71	2523.50	0.64
	M	69		
Interpreting emotion scenario 4	F	71	1930.50	0.04^a^
	M	69		
Dealing with emotion scenario 4	F	71	2354.50	0.96

a Significant values for p < 0.05.

**Table 6:** Mann Whitney test results for sex and strategies for interpreting emotional experience.

Variables	Sex	N	U	*P*
Selection of the situation - approach	F	71	1947.00	0.35^a^
	M	69		
Modifying the situation	F	71	1770.50	0.00^a^
	M	69		
Modifying attention focusing – rumination	F	71	2667.50	0.11
	M	69		
Cognitive restructuring	F	71	1774.50	0.11
	M	69		

Note: N = 143.

a Significant values for p < 0.05.

This means that integrating the information from what was said in the interviews, girls used these strategies more than boys. Girls realized that something was bothering them (e.g., “When I saw this post, I realized that something bothered me”), and then realized what was going on, in order to help (e.g., “I tried to understand what was going on to feel able to help.”), by reading the posts several times to better interpret the incident (e.g., “I read the post several times to understand what was going on”).

To verify the influence of the sex variable on emotions and emotion regulation strategies, the Mann-Whitney non-parametric test was performed, and the results showed that the sex variable influenced unpleasant emotions, in the sense that girls scored higher than boys (see Table 5). There was also an influence on the emotional regulation strategy interpretation of the emotional experience, with higher scores for girls (see Table 6) through the strategies of selecting the situation (approach) and modifying the situation.

**Fig. 5:**
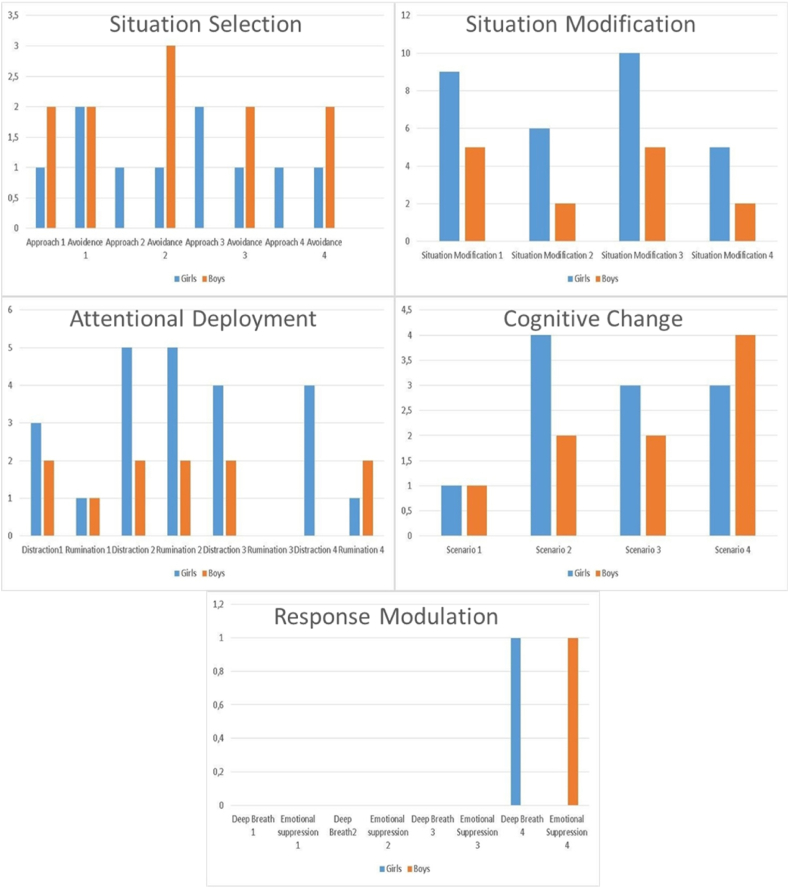
All emotional regulation strategies used by boys and girls in different cyberbullying scenarios.

From the qualitative analysis, it was possible to analyze that boys used the avoidance emotion regulation strategy (P27*: “I did nothing”*) more than girls, with the exception of scenario 1, which was considered by both as being more serious. Girls were more likely to try and understand what was happening in order to help (P2: *“Report or tell others that it was wrong”*), which corroborated our quantitative results found (see Fig. 5). Posting pictures and insults of a sexual nature in the online context had a greater impact on both boys (P6: “*I sent a message in the chat room and then said that we should talk to both of them calmly and calm Tatiana and Nando down*”) and girls, using an emotion regulation strategy of modifying the situation, helping to solve it and protecting the victim.

Compared to boys, girls were more likely to use the emotion regulation strategy of modifying the situation in all scenarios, which is in line with the quantitative results found. In all scenarios girls were those who most used the distraction emotion regulation strategy to deal with the emotion that the scenario provoked in them. Girls used distraction more in scenario 2 and not in scenario 1, as it was considered something more serious and for which intervention is needed. Both girls and boys perceived scenario 2 as the one they saw as being most common in their daily lives and were therefore, more used to seeing these situations (insults). The girls felt more uncomfortable than the boys with the insults they see in their daily lives, using the emotion regulation strategy of distraction more often (P1: “*I saw it, I was a little bit ready, but then I didn't think about it anymore and left it”*), and also considered the situation more serious than the boys (P2: “*I didn't feel emotion, it's something I'm used to*”).

In scenario 2, girls used the emotion regulation strategy cognitive change more, whereas boys used this strategy more in scenario 4 (see Fig. 5). Boys assessed the danger in scenario 4 by devaluing what was happening, giving it a new interpretation, based on humor, which may have been more related to differences in personality characteristics between sexes and in the way they analyzed situations (P6: “*I don't know how he's more of a joke guy, maybe he wouldn't take the situation so seriously”*). They also tended to not take situations so seriously (P11: “*they don't always do what they say”*). In scenario 2, the girls also tended to devalue the situation considering the insults and threats as fake (P9: “*I thought it was bluff”*); (P10: “*nobody threatens someone like that over the internet”, “I know it's not real”*), (P14: “*it was a bit exaggerated on her part, false”*). The emotion regulation strategy modulation of emotional response (deep breathing and emotional suppression) (see Fig. 5), was not used frequently by either boys or girls. This type of strategy was only reported in scenario 4, which referred to the threat of posting personal content without authorization, contrarily to the quantitative results, where there was a higher frequency of use of these strategies, with the emotion regulation strategy suppression of emotions being used more by boys than girls.

### 3.4 Differences between seventh and eighth graders in terms of emotions and emotion regulation in cyberbullying

Regarding the grade variable, in Table 7 results showed that it had an effect on the pleasant emotions felt in scenario 2 (*U* = 2092.5000; *P* < 0.05), scenario 3 (*U* = 2125.000; *P* = 0.027) and no effect in scenario 1 (*U* = 2038.500; *P* > 0.05) and scenario 4 (*U* = 2350.500; *P* < 0.504). This means that seventh graders experienced pleasant emotions when they observed the scenarios that referred to physical threats or homophobic comments. However, there were no differences in the scenarios referring to posting photographs, spreading rumors and threats with someone else's personal information.

**Table 7:** Mann-Whitney test results for schooling, emotions and regulation strategies variable.

Variables	Grade	N	U	*P*
	7th	68	2308.50	0.39
	8th	72		
Unpleasant emotions scenario 1	7th	68	2209.50	0.31
	8th	72		
Pleasant emotions scenario 2	7th	68	2092.50	0.01^a^
	8th	72		
Unpleasant emotions scenario 2	7th	68	2044.50	0.89
	8th	72		
Pleasant emotions scenario 3	7th	68	2125.00	0.02^a^
	8th	72		
Unpleasant emotions scenario 3	7th	68	1873.50	1.16
	8th	72		
Pleasant emotions scenario 4	7th	68	2350.50	0.50
	8th	72		
Unpleasant emotions scenario 4	7th	68	2025.00	0.07
	8th	72		
Interpreting emotion scenario 1	7th	68	2710.00	0.19
	8th	72		
Dealing with emotion scenario 1	7th	68	2138.50	0.84
	8th	72		
Interpreting emotion scenario 2	7th	68	2543.50	0.69
	8th	72		
Dealing with emotion scenario 2	7th	68	2006.50	0.08
	8th	72		
Interpreting emotion scenario 3	7th	68	2422.50	0.96
	8th	72		
Dealing with emotion scenario 3	7th	68	1977.50	0.06
	8th	72		
Interpreting emotion scenario 4	7th	68	2278.00	0.56
	8th	72		
Dealing with emotion scenario 4	7th	68	1687.00	0.00^a^
	8th	72		

a Significant values for p < 0.05.

In Table 8 the results showed that the school grade had an effect on pleasant emotions, namely, the emotion “pride” in scenarios 2 (*U* = 1873.500; *P* < 0.005), 3 (*U* = 1873.500; *P* < 0.05) and scenario 4 *(U* = 2223.000; *P* < 0.036). The results also showed that school grades had an effect on emotion regulation strategies. Since in Table 7 the results show that school grade had an effect on emotion regulation coping strategies, the Mann-Whitney test was performed to check the effect of school grade on emotion coping strategies in the 4 scenarios. The results obtained indicated that there was an effect of the school grade variable on the regulation strategy “emotion suppression” in scenario 1 (*U* = 1980.500; *P* < 0.05), with an overlap of seventh grade. This means that seventh grade students resorted to this emotion regulation strategy to cope with the observed emotional experience. The same effect holds for scenario 2 (*U* = 1837.000*; P* < 0.05), scenario 3 (*U* = 1837.000; *P* < 0.05) and scenario 4 (*U* = 1604.500; *P* < 0.05). In scenario 3 and 4, the results showed an effect of the grade variable on distraction emotion regulation strategies (*U* = 1937.000; *P* < 0.05; *U* = 1844.000; *P* < 0.05) (see Table 9). Responses for these two variables were higher in seventh grade. In sum, the results indicated that for the total sample, scenario 1 followed by scenario 3 were those that had the greatest impact on adolescents.

**Table 8:** Mann-Whitney test results for school grade variable for pleasant emotions.

Variables	Grade	N	U	*P*
Happy scenario 1	7th	68	2427.00	0.86
Happy scenario 1	8th	72		
Enthusiastic scenario 1	7th	68	2428.00	0.89
Enthusiastic scenario 1	8th	72		
Proud scenario 1	7th	68	2254.50	0.84
Proud scenario 1	8th	72		
Happy scenario 2	7th	68	2190.50	0.54
Happy scenario 2	8th	72		
Enthusiastic scenario 2	7th	68	2078.00	0.38
Enthusiastic scenario 2	8th	72		
Proud scenario 2	7th	68	1873.50	0.00^a^
Proud scenario 2	8th	72		
Happy scenario 3	7th	68	2297.00	0.11
Happy scenario 3	8th	72		
Enthusiastic scenario 3	7th	68	2213.00	0.07
Enthusiastic scenario 3	8th	72		
Proud emotion scenario 3	7th	68	2081.50	0.00^a^
Proud emotion scenario 3	8th	72		
Happy scenario 4	7th	68	2506.50	0.54
Happy scenario 4	8th	72		
Enthusiastic scenario 4	7th	68	2329.00	0.38
Enthusiastic scenario 4	8th	72		
Proud scenario 4	7th	68	2223.00	0.03^a^
Proud scenario 4	8th	72		

a Significant values for p < 0.05.

**Table 9:** Results of the Mann-Whitney test for school grade and emotion regulation strategies to deal with emotions.

Variables	Grade	N	U	*P*
Selection of the Situation -distraction in scenario 1	7th	68	2263.00	0.42
Selection of the Situation -distraction in scenario 1	8th	72		
Cognitive Change in scenario 1	7th	68	2681.00	0.32
Cognitive Change in scenario 1	8th	72		
Emotional Suppression in scenario 1	7th	68	1980.50	0.04
Emotional Suppression in scenario 1	8th	72		
Selection of the Situation -Avoidance in scenario 2	7th	68	2352.00	0.64
Selection of the Situation -Avoidance in scenario 2	8th	72		
Selection of the Situation -distraction in scenario 2	7th	68	2046.50	0.07
Selection of the Situation -distraction in scenario 2	8th	72		
Cognitive Change in scenario 2	7th	68	2644.50	0.40
Cognitive Change in scenario 2	8th	72		
Emotional Suppression in scenario 2	7th	68	1837.00	0.01^a^
Emotional Suppression in scenario 2	8th	72		
Selection of the Situation -Avoidance in scenario 3	7th	68	2205.50	0.21
Selection of the Situation -Avoidance in scenario 3	8th	72		
Selection of the Situation -distraction in scenario 3	7th	68	1937.00	0.02^a^
Selection of the Situation -distraction in scenario 3	8th	72		
Cognitive change in scenario 3	7th	68	2388.50	0.80
Cognitive change in scenario 3	8th	72		
Emotional Suppression in scenario 3	7th	68	1963.00	0.03^a^
Emotional Suppression in scenario 3	8th	72		
Selection of the Situation -Avoidance in scenario 4	7th	68	2205.50	0.21
Selection of the Situation -Avoidance in scenario 4	8th	72		
Selection of the Situation -distraction in scenario 4	7th	68	1844.00	0.00^a^
Selection of the Situation -distraction in scenario 4	8th	72		
Cognitive Change in scenario 4	7th	68	2352.00	0.79
Cognitive Change in scenario 4	8th	72		
Emotional Suppression in scenario 4	7th	68	1604.50	0.00^a^
Emotional Suppression in scenario 4	8th	72		

a Significant values for p < 0.05.

The emotion regulation strategies interpretation of emotional experience for the grade level variable were the most present in scenarios 1, 2, and 3 (as exemplified in Table 7). The emotion regulation strategies used to interpret the emotion were situation selection (approach), rumination (about the victim's feelings), and cognitive change. These strategies had the highest value in scenario 1, with the exception of cognitive change (putting yourself in the victim's place), which revealed the highest frequency of responses in scenario 3. Of the coping strategies, situation selection (rumination), cognitive change (putting yourself in the place of the victim or aggressor), and emotional suppression were more frequent in scenarios 1 and 3.

The school grade variable showed an effect on pleasant emotions for seventh graders, with higher scores for the emotion pride in scenarios 2 and 3 and an effect on the emotion regulation strategy of dealing with the emotional experience through the emotion regulation strategies of emotional suppression in all scenarios, as well as situation selection (distraction) in scenarios 3 and 4 (see Table 9).

## 4 Discussion

The study acknowledges the potential variation in emotional responses and the choice of emotion regulation strategies among bystanders in response to different forms of cyberbullying. Recognizing the importance of analyzing potential sex differences among adolescents in their approach to emotion regulation in cyberbullying, the research aims to determine whether gender plays a significant role in shaping the interventions needed [95,96]. To enhance the discussion and interpretation of results, the research questions are designed to explore various cyberbullying scenarios. These include examining the emotions and emotion regulation strategies adopted by bystanders, analyzing potential differences between boys and girls in their emotional responses and regulation strategies, and investigating whether disparities exist between seventh-grade and eighth-grade students in terms of their emotional experiences and adopted emotion regulation strategies.

### 4.1 Emotions for different scenarios of cyberbullying

In addressing the first research question regarding the emotions and emotion regulation strategies of cyberbullying bystanders in various observed aggressive scenarios, quantitative findings revealed that scenarios 1 (publication of the victim's picture with offensive comments) and 3 (homophobic comments) triggered more unpleasant emotional responses in adolescents. Specifically, adolescents frequently reported feeling anger, followed by worry and sadness. These quantitative results aligned with qualitative data, highlighting scenario 1 as the most impactful on adolescents, consistent with existing literature [[26], [27], [28]].

One potential explanation for these results is that adolescents may perceive the publication of photographs as more harmful due to the private nature of the content becoming public, exposing the victim to ridicule on a larger scale. Authors like Slonje and Smith [26] suggested that victims not knowing who viewed the photograph generates fear and anxiety. In contrast, scenarios involving threats of physical aggression (scenario 2) and sharing the victim's personal information without permission to spread rumors (scenario 4) had a lesser impact in both quantitative and qualitative analyses.

This diminished impact might be linked to the commonality of witnessing threats to physical integrity or observing others making threats, leading adolescents to be desensitized to such behaviors. This desensitization, documented in the literature [14,97], suggests that the frequency of exposure to cyberbullying may influence perceived severity. Studies supporting this notion indicate insults or threats as the most frequent types of cyberbullying, followed by image appropriation. The infrequency of scenario 1 may explain its heightened negative emotional response, lacking desensitization and therefore perceived as more severe.

Qualitative results consistently highlighted anger as the predominant emotion across all scenarios, with scenario 1 evoking it most frequently and scenario 3 eliciting sadness. Emphasizing the importance of studying emotion revolt, closely related to anger, existing literature identifies anger as a predictor of cyberbullying behavior [58]. This underscores the significance of exploring emotional reactions to cyberbullying scenarios and their potential implications for intervention strategies.

### 4.2 Emotional regulation strategies for different scenarios of cyberbullying

The statistical analysis conducted with ERAC categorized James Gross' emotion regulation model into two primary strategies: interpreting the emotional experience and coping with the emotional experience. The quantitative data revealed that interpreting emotional experience strategies were most prevalent in scenarios 1, 2, and 3, and least frequent in scenario 4. The most frequently used strategies for interpreting emotions were situation selection, rumination on the victim's feelings, and cognitive change, with scenario 1 having the highest prevalence, except for cognitive change, which was more frequent in scenario 3.

Adolescents predominantly employed strategies directed towards interpreting the emotional experience, considered positive for pro-social behavior. Coping strategies, including situation selection (rumination), cognitive change (putting oneself in the place of the victim or aggressor), and emotional suppression, were more prevalent in scenarios 1 and 3. This suggests that, across all scenarios, adolescents sought to interpret their emotions to act on the emotions felt by understanding the severity of the situation. They engaged in activities like repeatedly reading posts, analyzing messages, empathizing with the victim, and placing themselves in the victim's shoes, intending to provide assistance [98].

Contrary to literature suggesting rumination as a maladaptive strategy, the results indicated a positive context in this cyberbullying scenario. Rumination was associated with increased empathy in bystanders, contributing to pro-social behavior among adolescents. Spending more time contemplating the situation may enhance the likelihood of effective intervention and assistance to the victim, contradicting the expected negative outcomes.

Emotion regulation strategies to deal with the emotional experience, particularly emotional suppression, were reported in all four scenarios, with the highest prevalence in scenarios 1 and 3. Emotional suppression, focused on the observer's emotional management, may be considered maladaptive, lacking social support for the victim. Emotional suppression in scenarios triggering the greatest emotional impact suggests potential maladaptive coping mechanisms. This may be related to peer pressure, a reluctance to disclose one's feelings, or suppressing positive emotions due to concerns about peer acceptance.

Qualitative findings indicated that some adolescents refrained from providing social support to the victim, deeming the situation unserious or not involving someone within their close friendship circle. This could further explain the preference for emotional suppression as a coping strategy. Overall, these results underscore the complexity of adolescents' emotional and coping responses in cyberbullying scenarios and highlight the need for nuanced intervention strategies [[62], [66], [99], [100], [101]].

### 4.3 Differences between boys and girls in the emotions felt and the adopted emotion regulation strategies

Addressing the second research question – examining gender differences in emotions felt and emotion regulation strategies adopted when facing various cyberbullying scenarios – the quantitative findings revealed that, in general, girls experienced more negative emotions than boys. Moreover, girls tended to utilize the emotion regulation strategy of interpreting the emotional experience, specifically through strategies such as situation recognition, situation selection (approach), and situation modification, more frequently than boys. These results align with existing literature indicating that girls often report more psychological symptoms, exhibit greater vulnerability to stressful life events, and demonstrate heightened sensitivity to others' problems [102,103]. Additionally, girls are commonly identified as more pro-social, a trait consistent with the observed results [98].

However, in scenario 1, both boys and girls did not exhibit significant differences in terms of negative emotions. This suggests that, for boys, the act of posting photos was perceived as more serious, capable of evoking stronger negative emotions like anger or sadness, compared to scenarios involving online threats and homophobic insults – although girls were still sensitive to these situations. These findings are in harmony with research among adults [104], indicating that content like photos or videos with highly aggressive material elicits more pronounced negative emotional responses. This underscores the context and nature of aggression as critical factors influencing emotional responses.

Conversely, the observation that boys reported fewer negative emotions than girls in other scenarios may be attributed to desensitization. This phenomenon could be linked to the communication styles commonly employed by boys, who often use more direct and aggressive forms of communication (e.g., insults, physical aggression) compared to girls, who tend to utilize more indirect forms (e.g., social exclusion). These gender-specific patterns in emotional responses emphasize the nuanced nature of cyberbullying scenarios and the importance of considering individual communication styles and contextual factors [104].

### 4.4 Differences between seventh and eighth grades for the emotions felt and the adopted emotion regulation strategies

The third and final research question aimed to explore variations between seventh and eighth-grade students concerning their emotions and emotion regulation strategies in different cyberbullying scenarios. Results indicated that the school grade variable influenced pleasant emotions for seventh graders, showing higher scores for pride emotion in scenarios 2 and 3 compared to scenario 1. This suggests that younger students might struggle in handling aggressive situations with high-intensity emotional responses, potentially due to more impulsive behavior and a lack of empathy, consistent with previous studies [8,13].

Moreover, the school grade variable had an impact on the emotion regulation strategy of “coping with emotional experience,” particularly through the strategies of “emotional suppression” in all scenarios and “situation selection” (distraction) in scenarios 3 and 4. Similar to victims of cyberbullying, bystanders exhibited a prevalent use of emotion-focused coping skills, aligning with emotional suppression. While distraction is considered adaptive for victims, for bystanders, it may indicate a failure to adopt pro-social behavior and support the victims [62]. The study did not account for whether adolescents had been victims themselves, potentially influencing these results and warranting further investigation in this area.

In summary, seventh graders experienced more pleasant emotions than eighth graders in scenarios 2 and 3, manifested through pride. Younger students seemed to value peer opinions more, potentially leading them to suppress positive emotions to conform to perceived social norms. Emotional suppression was employed for both positive and negative emotions, suggesting a potential impact on interpersonal relationships and overall well-being, as noted by Gross [104]. Additionally, adolescents utilized distraction strategies in scenarios 3 and 4, possibly as a way to emotionally distance themselves from continuous exposure to aggressive behavior.

This study contributes valuable insights to the literature, highlighting that even a one-year difference can significantly influence how young individuals emotionally react to cyberbullying scenarios and manage their emotions. This contrasts with some previous findings, emphasizing the importance of considering age as a variable in understanding online aggressive behavior, especially during early adolescence when patterns may shift [105,106].

### 4.5 Limitations and future research

The study's innovative use of new technologies for investigating bystander behavior and the integration of qualitative and quantitative methodologies were notable [106,107]. Discrepancies between quantitative and qualitative results raised concerns about potential social desirability effects in interviews, emphasizing a limitation. To address this, future research should consider rephrasing questions, ensuring anonymity, and introducing unrelated questions within the questionnaire. Item Response Theory is suggested to confirm response reliability and randomness [106,107].

This study's limitations included a limited sample size and age range, which could impact the generalizability of findings [106,107]. Future studies could expand the sample size, encompass a wider age range, and ensure greater ecological validity [106,107]. Moreover, it is crucial to acknowledge potential biases and constraints that may influence the interpretation of findings. The possibility of sample selection bias could occur. The study's sample, consisting of adolescents, may not be fully representative of the broader population, potentially limiting the generalizability of the results.

Furthermore, participants may provide responses influenced by social desirability or subjective interpretations of their emotional experiences. This may lead to a potential bias in reporting emotions. This may affect the accuracy of the information gathered, emphasizing the need for cautious interpretation when relying on self-reported data. We tried to mitigate these biases through rigorous research methodologies. However, these considerations should be kept in mind when drawing conclusions from the study's findings.

The study acknowledged the limitation of not examining the simultaneous use of emotion regulation strategies for interpreting and dealing with emotions among adolescents [106,107]. Future investigations should prioritize understanding these dual strategies to provide a nuanced comprehension of emotional responses in cyberbullying contexts [106,107].

The study did not explore variables such as impulsivity and empathy, which could contribute to understanding differences among younger and older participants [13]. Future research should incorporate these variables to enhance the understanding of emotion regulation dynamics in the context of cyberbullying [13].

Furthermore, the study highlighted a need for increased awareness among boys regarding various forms of aggressive behavior and normative online conduct [108]. Future research should delve into understanding how boys' emotion regulation strategies impact their responses to cyberbullying, considering potential positive and negative consequences. Replicating the study across diverse age groups, cultures, and regions is recommended [13,[106], [107], [108]].

### 4.6 Practical implications

This study serves as a crucial exploration into the emotional experiences of adolescents witnessing diverse cyberbullying scenarios, shedding light on an underexplored area, particularly from the bystander perspective. The utilization of self-reporting to investigate these emotions enhances the value of future interventions, offering a nuanced understanding that could inspire further studies and the development of targeted programs for young individuals. Based on the study's findings, there is a critical need to formulate and implement school-based intervention programs aimed at equipping adolescents with effective emotion regulation strategies to address cyberbullying [78,109]. These programs should be designed with a sensitivity to sex differences, school grade, and age. Specifically, interventions could focus on cultivating empathy and enhancing impulsivity control, particularly targeting younger boys. Additionally, efforts should be directed at reducing desensitization to aggressive behavior, addressing this issue comprehensively across various age groups [78,109]. Future interventions could emphasize teaching students pro-social and effective methods for handling emotions. Encouraging bystanders to support the victim and avoid engaging in bullying behaviors themselves is crucial [78,109]. In fact, tailoring interventions for specific grade levels, such as the distinction between seventh and eighth-grade students, is recommended. The latter group appears to exhibit emotional reactions and regulation strategies indicative of more pro-social behavior towards cyberbullying [78,109].

Recognizing the evolving landscape of technology use among adolescents, interventions could incorporate digital platforms to promote pro-social behavior. Leveraging technology in interventions can enhance motivation among bystanders to intervene adaptively, aligning with the realities of their experiences [110]. Further attention is required for future research focusing on using new technologies to encourage pro-social behavior in adolescents, thereby enhancing the reliability and ecological validity of interventions [110]. Considering the identified differences between genders and age groups in emotion regulation strategies, interventions, including educational games like the C@mViver game, should be tailored accordingly. For instance, the game can be designed to prompt student reflection on their behavior and its consequences for victims, aligning with specific emotion regulation strategies identified during gameplay [110].

These recommendations emphasize the imperative nature of context-aware interventions that leverage technology and address the unique emotional needs of adolescents. Tailoring programs to specific demographics, refining strategies based on gender and age differentials, and incorporating innovative tools like educational games are crucial steps towards fostering pro-social behavior and effective emotion regulation in the context of cyberbullying.

## 5 Conclusion

This study offers significant insights into how adolescents respond to various forms of cyberbullying, shedding light on both their emotional reactions and emotion regulation strategies. Notably, the findings highlight differences in emotional responses to different forms of aggression. Both boys and girls exhibit heightened emotional responses when confronted with the posting of victim pictures, deeming it a more serious form of aggression. Additionally, homophobic posts against the victim evoke more unpleasant emotional reactions, specifically in girls. Girls' inclination towards pro-social behavior further emphasizes the need for gender-specific considerations in future interventions.

A noteworthy outcome pertains to the substantial disparity between seventh-grade and eighth-grade students in their reactions to aggressive behavior. Seventh graders demonstrate a higher reliance on emotion regulation strategies involving emotional suppression (across all scenarios) and distraction (in scenarios 3 and 4). This suggests that younger students may face challenges in emotion regulation and emotional maturity, potentially elevating their vulnerability to developing cyberbullying behavior. However, fostering emotion understanding and regulation in youth may empower them to navigate cyberbullying experiences with greater resilience and integrity.

## Ethics statement

This research includes experiments with human participants, so authorizations were requested and collected from the Portuguese Ministry of Education, the Portuguese National Commission for Data Protection, the Ethics and Deontology Committee of the Faculty of Psychology of the University of Lisbon with approval and authorization number 5417/2016.The Director of a public school cluster in the Lisbon region, the teachers, the parents and the adolescents themselves have given their informed consent for the realization of this research.

## Data availability statement

Data associated with this study was not been deposited into a publicly available repository but will be made available on request. Data is confidential due to the nature of the study and was an obligatory requirement of the Faculty of Psychology of the University of Lisbon's Ethics Committee, whereas material is available upon request.

## Funding

This work was supported by 10.13039/501100001871FCT – Fundação para a Ciência e Tecnologia, I.P **(**http://doi.org/10.54499/PTDC/PSI-GER/1918/2020**)** and by the Research Center for Psychological Science of the Faculty of Psychology, and 10.13039/501100005765University of Lisbon [UIDB/04527/2020; UIDP/04527/2020].

## Consent

Authors’ give their consent for the article to be published.

## CRediT authorship contribution statement

**Sónia da Silva Gomes:** Writing – review & editing, Writing – original draft, Methodology, Investigation. **Paula da Costa Ferreira:** Supervision, Conceptualization. **Nádia Pereira:** Visualization. **Ana Margarida Veiga Simão:** Visualization, Supervision, Methodology, Investigation, Formal analysis, Conceptualization.

## Declaration of competing interest

The authors declare that they have no known competing financial interests or personal relationships that could have appeared to influence the work reported in this paper.

## Figures and Tables

**Table A.1:** Descriptive statistics and correlations

Items		Factor Loadings		Means	Correlations																
					1	2	3	4	5	6	7	8	9	10	11	12	13	14	15	16	17
1	I felt sad.		0.94	2.77 (1.69)																	
2	I felt revolted.		0.96	2.99 (1.81)	0.86																
3	I felt avenged.		0.46	1.97 (1.18)	0.52	0.54															
4	I felt happy.	0.64		1.66 (0.73)	0.41	0.44	0.42														
5	I felt defenseless.		0.59	1.95 (1.07)	0.62	0.56	0.54	0.39													
6	I was afraid.		0.66	1.97 (1.10)	0.65	0.6	0.45	0.42	0.6												
7	I was enraged.		0.97	2.64 (1.70)	0.76	0.83	0.59	0.41	0.58	0.57											
8	I felt indifferent.	0.50		1.98 (1.18)	0.29	0.33	0.28	0.41	0.36	0.34	0.27										
9	I felt secure.	0 0.82		1.96 (1.23)	0.39	0.4	0.36	0.47	0.37	0.28	0.41	0.39									
10	I felt proud.	0.80		1.63 (0.73)	0.39	0.38	0.35	0.52	0.38	0.37	0.32	0.32	0.56								
11	I felt angry.		0.95	2.72 (1.71)	0.76	0.82	0.52	0.4	0.58	0.55	0.82	0.33	0.42	0.41							
12	I felt anguished.		0.79	2.22 (1.40)	0.69	0.71	0.49	0.32	0.57	0.55	0.68	0.35	0.37	0.39	0.74						
13	I felt strong.	0.76		1.82 (1.00)	0.44	0.46	0.45	0.46	0.44	0.35	0.45	0.39	0.64	0.53	0.43	0.47					
14	I felt threatened.		0.62	1.96 (1.16)	0.58	0.55	0.45	0.32	0.57	0.62	0.55	0.26	0.33	0.44	0.59	0.66	0.43				
15	I felt enthusiastic.	0.82		1.73 (0.86)	0.41	0.42	0.4	0.49	0.37	0.3	0.38	0.4	0.56	0.59	0.45	0.46	0.59	0.43			
16	I felt irritated.		0.98	2.63 (1.67)	0.74	0.79	0.52	0.36	0.56	0.54	0.82	0.29	0.41	0.37	0.87	0.71	0.46	0.6	0.37		
17	I felt embarrassed.		0.55	1.88 (1.02)	0.57	0.56	0.52	0.43	0.59	0.54	0.53	0.31	0.41	0.41	0.59	0.61	0.46	0.69	0.43	0.62	
18	I was worried.		0.79	2.79 (1.75)	0.75	0.76	0.44	0.38	0.55	0.54	0.72	0.32	0.42	0.45	0.74	0.69	0.49	0.57	0.46	0.74	0.58
Eigenvalues		9.32																			

## Abbreviations

GC1: Control Group 1
GC2: Control Group 2
ECA: Emotions in Cyberbullying in Adolescents questionnaire
ERAC: Emotion Regulation of Adolescents in Cyberbullying questionnaire
IOCI: Inventory of Observed Cyberbullying Incidents
KMO: Kaiser-Meyer-Olkin
